# Identification of Late Embryogenesis Abundant (LEA) Protein Putative Interactors Using Phage Display

**DOI:** 10.3390/ijms13066582

**Published:** 2012-05-29

**Authors:** Rekha Kushwaha, Taylor D. Lloyd, Kim R. Schäfermeyer, Santosh Kumar, Allan Bruce Downie

**Affiliations:** 1 Department of Horticulture, University of Kentucky, Lexington, KY 40546-0312, USA; E-Mails: kushwaharekha@gmail.com (R.K.); taylor.d.lloyd@gmail.com (T.D.L.); ksc223@uky.edu (K.R.S.); santosh.nipgr@gmail.com (S.K.); 2 Seed Biology Group, University of Kentucky, Lexington, KY 40546-0312, USA

**Keywords:** secondary dormancy, seed germination, heat stress, LEA proteins

## Abstract

*Arabidopsis thaliana* seeds without functional SEED MATURATION PROTEIN1 (SMP1), a boiling soluble protein predicted to be of intrinsic disorder, presumed to be a LATE EMBRYOGENESIS ABUNDANT (LEA) family protein based on sequence homology, do not enter secondary dormancy after 3 days at 40 °C. We hypothesized that SMP1 may protect a heat labile protein involved in the promotion of secondary dormancy. Recombinant SMP1 and *Gm*PM28, its soybean (*Glycine max*), LEA4 homologue, protected the labile GLUCOSE-6-PHOSPHATE DEHYROGENASE enzyme from heat stress, as did a known protectant, Bovine Serum Albumin, whether the LEA protein was in solution or attached to the bottom of microtiter plates. Maintenance of a biological function for both recombinant LEA proteins when immobilized encouraged a biopanning approach to screen for potential protein interactors. Phage display with two Arabidopsis seed, T7 phage, cDNA libraries, normalized for transcripts present in the mature, dehydrated, 12-, 24-, or 36-h imbibed seeds, were used in biopans against recombinant SMP1 and *Gm*PM28. Phage titer increased considerably over four rounds of biopanning for both LEA proteins, but not for BSA, at both 25 and at 41 °C, regardless of the library used. The prevalence of multiple, independent clones encoding portions of specific proteins repeatedly retrieved from different libraries, temperatures and baits, provides evidence suggesting these LEA proteins are discriminating which proteins they protect, a novel finding. The identification of putative LEA-interacting proteins provides targets for reverse genetic approaches to further dissect the induction of secondary dormancy in seeds in response to heat stress.

## 1. Introduction

The Late Embryogenesis Abundant (LEA) proteins were first identified [1], and then named [2], from studies of cotton seed proteins, and homologues have been identified in a variety of organisms within and outside the plant kingdom in the ensuing 30 years [3,4]. A variety of LEA protein classification schemes exist and consensus is that these proteins are a sub-group of a larger family of proteins typified by an intrinsically disordered structure when fully hydrated [5,6]. This phenomenon, in combination with their high hydrophilicity, has been used to suggest that LEA proteins may act in a variety of ways to replace water (or compensate for its loss) in dehydrating tissues [6]. Regardless of the sequence diversity embodied within the LEA protein super families [4,7], hyperproduction of these proteins within the same or unrelated species can result in greater than usual tolerance to abiotic stress [8–11]. Conversely, a decrease in the capacity of a species to produce certain LEA proteins can result in reduced fitness under specific stresses [12–15]. Furthermore, the capacity of a range of different LEA proteins to protect a diversity of labile molecules from sundry abiotic stresses *in vitro* has contributed to the perception that LEA proteins are indiscriminate in their protective function [16,17]. This perception has gained support with the finding that, in certain abiotic stresses, synergistic protein/catalytic protection by LEA proteins results from the addition of oligosaccharides [18]. Nevertheless, uncertainty exists regarding whether LEA proteins serve exclusively as general “spacer” molecules (“molecular shields”) that simply prevent protein aggregation upon water loss or if they can act as specific protectors of individual target molecules [7,19].

LEA proteins from a variety of species have been shown to provide protection to commercially available labile proteins (reporter enzymes) during exposure to supra-optimal temperatures, dehydration, or freezing, either alone [20–23] or acting synergistically with oligosaccharides [18]. To date however, there have been no reports of the identity of any LEA protein’s preferred protein interactor from any species. It is possible that this is because all LEA proteins are truly molecular shields and have no preference for particular proteins. However, an example of a specific protective capacity has recently been demonstrated for a LEA protein from Pea (*Pisum sativum*) which is able to increase the protection it affords membranes from dehydration and freezing the greater the membrane’s content of cardiolipin, a phospholipid found only in bacterial and mitochondrial membranes [24]. The mitochondrion is the organelle in which this LEA protein is located and its protective capacity was optimal in lipid membranes whose composition most closely resembled that found in seed mitochondria. These results have led the authors to suggest that this LEA protein’s capacity to protect, and seed mitochondrial membrane composition, share a structure/function co-adaptation that results in maximum protection for mitochondrial membranes in cells of seeds that are destined to dehydrate [24].

The SEED MATURATION PROTEIN1 (SMP1; AT3G12960) has a glycine content of 7%, insufficient to classify it as a “hydophilin” (of which LEA proteins are a member) according to criteria set down in ([3]; glycine content ≤8%; hydrophilicity index ≤1). Nevertheless, SMP1 has an amino acid sequence similarity closest to that of a protein found in *Glycine tomentella* which, in turn, is most similar to the LEA4 protein, *Gm*PM28 from *Glycine max* [25]. The two proteins differ in length by 3 amino acids (SMP1: 86aas; *Gm*PM28: 89aas), have a level of identity of 57%, and are 88% similar, So, despite SMP1 not being included as a LEA protein in either of the Arabidopsis surveys of these proteins [26,27], in this article, SMP1 and *Gm*PM28 will be referred to, in the aggregate, as LEA proteins.

When SMP1 is absent from mature Arabidopsis seeds due to T-DNA insertional mutagenesis, the resulting seeds of either the Columbia or Landsberg erecta ecotypes do not enter secondary dormancy upon exposure to supraoptimal temperatures [28]. One interpretation of this result is that SMP1 is a LEA protein that interacts with and protects some cellular component(s) required for seeds to enter secondary dormancy. Secondary dormancy is an adaptive trait arising in previously non-dormant seeds due to unfavorable environmental conditions during germination [29–31]. Secondary dormancy is useful in synchronizing the completion of germination with the re-establishment of conditions conducive for seedling establishment [32]. Assuming that the heat-labile, protected component was a protein, and using recombinant SMP1 and its soybean LEA protein homologue (*Gm*PM28) as bait, phage display cDNA libraries were used to pan for proteins to which the LEA proteins would bind at 25 and 41 °C to identify (a) protein(s) involved in inducing secondary dormancy in *Arabidopsis thaliana* due to heat stress. The T7 phage is particularly well suited to this study because the 41 °C experimental temperature is much lower than the published thermal maximum for T7 viability (~60 °C; [33]) and low enough not to promote selection of heat-stable mutant phage as false positives [34].

Biopanning with the two recombinant LEA protein homologues retrieved protein targets from two different libraries at two different temperatures. The putative LEA protein interactors were primarily categorized as “cytosolic” and were associated with the ribosome. The recurrent selection of at least three of these proteins (all of unknown function) provides potential targets to assess for involvement in secondary seed dormancy. This is the first time LEA proteins have been documented to be selective for the proteins to which they possibly bind, which is a novel finding for potential protein interactors akin to that described for the mitochondrial localized LEA protein for membrane phospholipids of a mitochondrial composition [24].

## 2. Results and Discussion

### 2.1. The Two LEA Proteins Are Boiling-Stable Proteins

**Figure 1:**
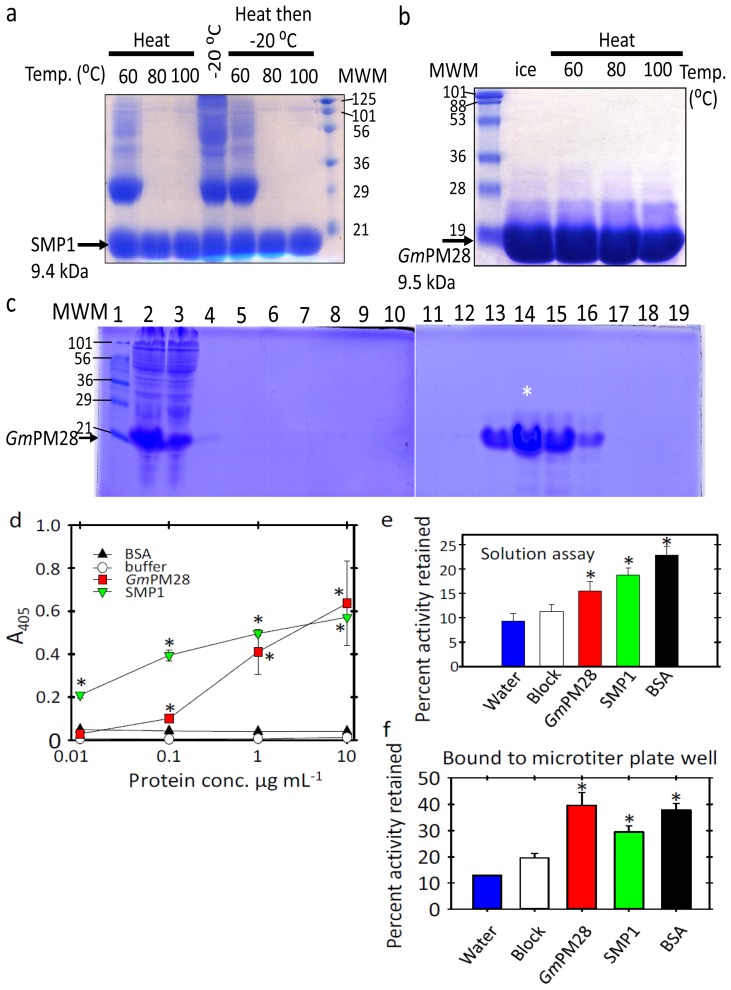
Recombinant protein expression and purification for use as biopanning bait. (**a**) Heated *E. coli* lysates of SMP1; (**b**) Heated fraction 15 GmPM28 recombinant protein; (**c**) Both SMP1 and *Gm*PM28 were recovered from lysed *E. coli* and the hexahistidyl tagged proteins purified on a nickel-charged column. SDS-PAGE (15%) separate the proteins in 10 μL of: (1) marker; (2) protein before filtration; (3) unbound proteins; (4) start of, and; (5) end of the column wash; (6–9) every third fraction; Lanes (10–16) fraction 11 through 17; lanes (17–19) fractions 20, 23 and 26; (**d**) ELISA assays showing the LEA proteins attach to the microtiter plate wells. Mean ± standard error at pH 7.5 depicted; (**e**) In solution G6PDH protection assays with LEA proteins, BSA, commercial blocking reagent or water; (**f**) G6PDH protection assays using various proteins or block bound to the microtiter plate well. Asterisks in **d**-**f** denotes significant differences among: (**d**) ELISA readings for SMP1 or GmPM28 and BSA/buffer; (**e**,**f**) percent activity retained relative to the block.

In keeping with a classification as hydrophilic LEA proteins [35,36] both SMP1 and *Gm*PM28 are boiling-stable proteins (Figure 1a,b). *E. coli* lysates of SMP1 were heated to 60, 80 or 100 °C for 10 min, centrifuged and the supernatant either boiled again in SDS-loading dye and electrophoresed directly (Figure 1a, Heat) or frozen at −20 °C for 12 h prior to boiling and SDS-PAGE analysis (Figure 1a, Heat then −20 °C). The middle lane (Figure 1a, −20 °C) was the lysate that was frozen for 12 h but not heated before centrifugation and SDS-PAGE. Ten-microliter of purified *Gm*PM28 fraction 15 (see * in Figure 1c) was subjected to SDS-PAGE analysis after 10 min on ice or at 60, 80, or 100 °C followed by centrifugation (Figure 1b).

Even though boiling resuspended *E. coli* pellets containing the recombinant proteins for 20 min provided a facile means of purifying large quantities of both LEA proteins (Figure 1a and data not shown), the proteins used in G6PDH protection assays and as bait for phage display were harvested from *E. coli* using lysozyme and freeze/thaw cycles followed by benzonase treatment and purification using nickel affinity. This was to minimize isoaspartyl (isoAsp) formation during exposure to heat [37], as SMP1 was identified as a protein that forms isoAsp and becomes a target of PROTEIN ISOASPARTYL METHYLTRANSFERASE1 [28]. Such post-translational modifications are frequently detrimental to protein function [38]. Using hexahistyl tagging of the recombinant protein and nickel-affinity chromatography (Figure 1c and data not shown), substantial amounts of both LEA proteins were purified. For example, the GmPM28 protein fractions 14–17 (lanes 13–16) were pooled and dialyzed against two 4 L changes of Tris, pH 7.5 at 4 °C and, upon dialysis, were used in G6PDH protection assays and as phage display bait.

### 2.2. Both LEA Proteins Afford Limited Protection to GLUCOSE-6-PHOSPHATE DEHYDROGENASE against Heat Stress

Prior to commencing biopanning, it was important to define a protective role for SMP1 and *Gm*PM28 toward other proteins under heat stress, particularly when bound to the bottom of microtiter plate wells. First, recombinant LEA proteins or BSA were diluted to 10 μg·mL^−1^ in either Tris or bicarbonate buffer (10 mM, pH was 7.5 (Tris-HCl), 9.6, or 10.6 (Carbonate)) and various concentrations introduced into microtiter plate wells. ELISA assays using the pentahistidyl tag of the recombinant proteins were performed and developed using a pentaHis primary antibody and an alkaline phosphatase-conjugated secondary antibody and *para*-nitrophenylphosphate substrate. ELISAs confirmed that the proteins were capable of binding to the microtiter plate wells. The *p*-values of the *t*-Tests for color intensity relative to that in the buffer containing wells was: SMP1 and GmPM28, respectively at, 10 μg·mL^−1^ (0.008 and 0.047), at 1 μg·mL^−1^ (0.001 and 0.007), at 0.1 μg·mL^−1^ (0.009 and 0.02) and at 0.01 μg·mL^−1^ (0.01 and 0.14 [ns]) (Figure 1d). Next, GLUCOSE-6-PHOSPHATE DEHYDROGENASE (G6PDH) was used as a labile protein known to lose activity upon heating, an activity which the general protective protein, BSA, was effective at preserving [39]. In solution-based assays of G6PDH activity, if GmPM28 was not present during heating, or if it was added after heating, before or after another 1 h at 41 °C, no protection was afforded to G6PDH and the activity retained was 12, 12, and 15% relative to that of newly thawed enzyme, respectively. Recombinant LEA proteins or BSA (all 530 pmol), block or water were added to G6PDH (144 pmol) and assayed immediately or after heating at 41 °C for 1 h. The percentage of activity retained after 41 °C (Mean ± standard error) was compared with that of unheated G6PDH (*i.e*., 100%). When G6PDH was heated for 1 h at 41 °C in the presence of water or commercial blocking reagent (EMD Chemicals Inc., Gibbstown, NJ, USA) enzyme activity tended to be less than when either LEA protein or BSA were present during heating, relative to the unheated G6PDH control (Figure 1e). The *p*-values of the *t*-Tests are: SMP1 (0.008), GmPM28 (0.032) and, BSA (0.007) (Figure 1e). When recombinant protein was attached to the bottom of microtiter plates and assayed for its capacity to protect G6PDH activity at 41 °C over 1 h, BSA, SMP1 and *Gm*PM28 preserved more G6PDH activity than the blocking reagent alone (Figure 1f). Percentages of G6PDH activity after 1 h at 41 °C (Mean ± standard error) when alone (water) or in Block-, BSA- (BSA), SMP1- (SMP1), or *Gm*PM28-coated (*Gm*PM28) wells were calculated relative to the enzyme assayed immediately after thawing (100%). The *p*-values of the *t*-Tests are: SMP1 (0.01), GmPM28 (0.02) and, BSA (0.005) (Figure 1f). These results suggest that the recombinant proteins were capable of some potentially nonspecific protection toward a heat labile enzyme and that they retained biological function even when bound to a solid support.

### 2.3. Phage Was Retained by Both LEA Proteins

**Figure 2:**
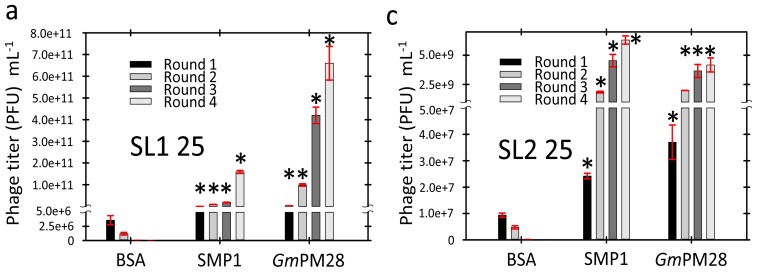

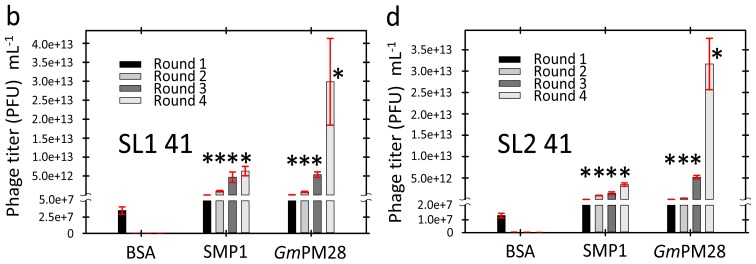
Phage titer (plaque forming units (PFU)·mL^−1^) at each of the four rounds of biopanning at 25 °C (**a** and **c**) and 41 °C (**b** and **d**) for seed library 1 (SL1; **a** and **b**) and SL2 (**c** and **d**). Asterisks over bars for SMP1 or *Gm*PM28 denote significant differences between titers for BSA relative to either SMP1 or *Gm*PM28 in each biopanning round. The *p*-values for SMP1 and GmPM28, respectively for rounds 1–4 (R1–R4) are: (**a**) all less than 0.01; (**b**) R1 0.02 and 0.005, R2 0.006 and 0.006, R3 0.03 and 0.002 and, R4 0.007 and 0.049; (**c**) R1 < 0.001 and 0.02, R2 both < 0.001, R3 0.001 and 0.002, R4 < 0.001 and 0.002; (**d**) all less than or equal to 0.01.

Regardless of the incubation temperature, if SMP1 or *Gm*PM28 was bound to the wells, phage titer increased during successive biopanning rounds though the final titer was greatest at the higher temperature. At the end of four rounds of biopanning, regardless of the library used, BSA was incapable of retaining phage; as observed by the steady decrease in phage titer. This demonstrated that the phage were not binding indiscriminately to the LEA proteins, the blocking agent used, or the well itself (Figure 2a–d). At 25 °C, the final phage titer differed significantly at the end of four rounds of biopanning between the two libraries with a much greater titer for seed library 1 (SL1) for both LEA proteins (Figure 2a *versus* 2b). This difference was not evident at 41 °C where final phage titer, although considerably greater than that attained after 4 rounds of biopanning at 25 °C, was remarkably similar between libraries (SMP1: ~5.0 × 10^12^ and *Gm*PM28: ~3.0 × 10^13^ for SL1 and SL2, respectively; Figure 2c,d, respectively).

### 2.4. LEA Protein-Retained Phage Contained Insert

The increase in titer over four rounds of biopanning was accompanied by an increase in the number of randomly selected phage containing substantially sized insert (randomly chosen cutoff of >300 bp equates to insert-containing) when round 1 was compared to round 4 for both libraries. For SL1 panned at 25 °C, both SMP1 and *Gm*PM28 increased the percentage of phage containing inserts from 39 to 83% (SMP1; Figure S.1a) and from 50 to 83% (*Gm*PM28; Figure S.1b). When panned over BSA at 25 °C, insert-containing phage declined from 28 to 0% (Figure S.1c). At 41 °C, SMP1 increased the percentage of insert-containing phage from 33 to 67% while *Gm*PM28 increased from 33 to 61% (Figure S.1d,e, respectively). BSA declined from 17 to 0% of randomly chosen phage containing insert greater than 300 bp (Figure S.1f). For SL2, both SMP1 (17% to 89%) and *Gm*PM28 (44% to 50%) increased the number of insert-containing phage when panned at 25 °C (Figure S.2a,b). BSA at 25 °C, on the contrary, reduced the number of phage that contained inserts over four rounds of biopanning (from 11% to 0; Figure S.2c). The increase in the number of phage that contained large inserts increased most dramatically when incubation with the LEA proteins occurred at 41 °C. For SMP1 the percentage increased from 17 to 89% and for *Gm*PM28, from 17 to 94% of randomly chosen plaque after four rounds of biopanning, as opposed to 22 to 0% for BSA (Figure S.2d–f, respectively).

### 2.5. The Two LEA Protein Homologues Target Discrete Proteins, Some of Which Are Identical

**Figure 3:**
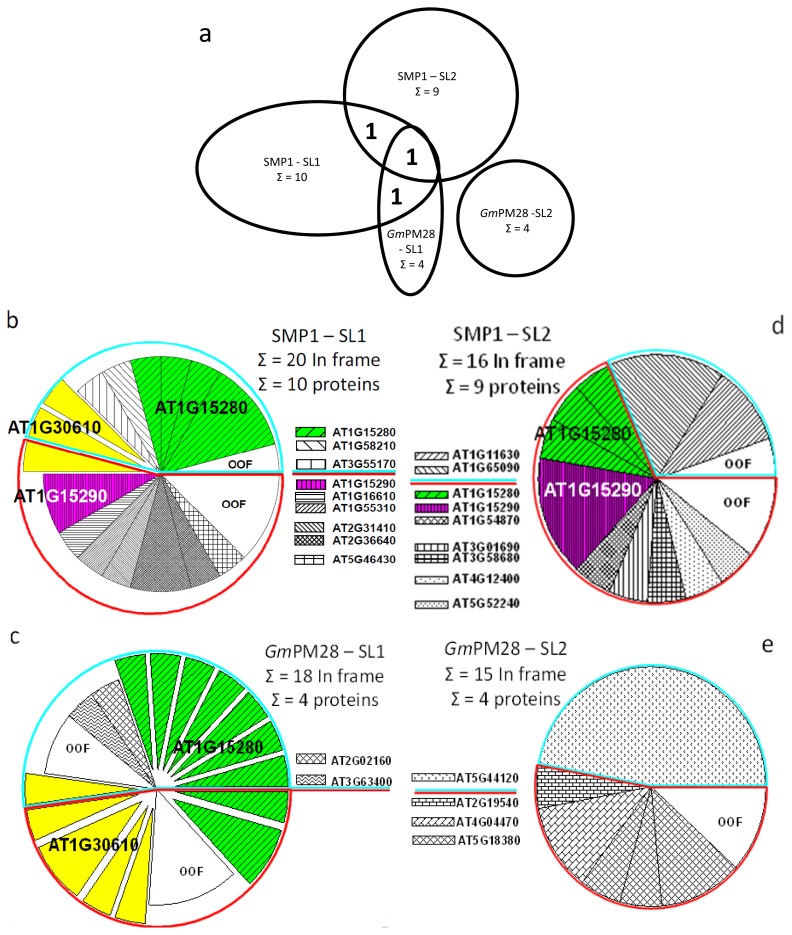
(**a**) A Venn diagram of the different proteins recovered by the different LEA proteins using the two different libraries; (**b**–**e**) Pie charts depicting the number of recovered hits that were in- and out-of-frame (OOF), the proteins identified, the number of identical clones for a portion of a protein recovered, and the number of independent clones encoding a portion of the same protein recovered. SMP1 (**b** and **d**) or *Gm*PM28 (**c** and **e**) were used in pans at 25 °C or 41 °C using seed library 1 (SL1; **b** and **c**) or SL2 (**d** and **e**).

By the end of four rounds, when the DNA from randomly selected phage containing insert were sequenced and compared, all pans contained far more in-frame proteins than would be expected through random chance from libraries that, by their design, contain 67% of their clones in the wrong frame (an average of 84% in-frame clones per pan and a range of 73% to 100%). Additionally, there were clones encoding portions of the same proteins that were repeatedly retrieved from pans at different temperatures, from the two different libraries, and/or by both LEA proteins. This is represented by the regions of intersection among the ellipses denoting potential protein targets in common to those pans (Figure 3a).

For all pie charts, exploded slices within a pie represent clones encoding portions of the same protein recovered from pans conducted at the two different temperatures. Demarcated slices of the same color within a pie constitute independent clones encoding different portions of the same protein. Each putative protein target is represented by a unique fill pattern and/or color to emphasize when clones encoding the same protein were recovered in independent libraries or by the two different LEA protein homologues (Figure 3b–e). However, OOF fills do not indicate identical OOF hits within or among pies (Figure 3b–e). Portions of a pie enclosed in a blue boundary are those that were recovered at 25 °C and those enclosed in a red boundary are for 41 °C (Figure 3b–e). Clone identities displayed above the blue line were recovered at 25 °C while those below the red line were recovered at 41 °C. The three hits repeatedly and independently recovered are indicated over the pies in which they occurred (Figure 3b–e). The number after the summation symbol indicates how many putative protein targets were identified in each set of pans (Figure 3a–e).

Because both recombinant proteins possess hexahistidyl tags whereas neither BSA nor the protein in the commercial blocking solution does, it was possible that the phage retained in the pans were bound by the tags and not the LEA proteins. However, when examining proteins susceptible to isoAspartate formation [28], none of the 17 in-frame targets retained by the recombinant, hexahistidyl tagged, PROTEIN ISOASPARTYL METHYLTRANSFERASE1 were recovered in the current screen using recombinant LEA proteins. The two screens were conducted using the same two libraries and some of the screens were done at the same temperature (25 °C). This rules out the hexahistidyl tag itself as being the cause of multiple, similar proteins being retained in the LEA protein screen. It is possible that the carboxy terminus of the LEA proteins and the hexahistidyl tag combined to capture the putative LEA protein interactors. However, the carboxy-termini of the two LEA protein homologues are their least homologous regions, sharing only 20% identity, 40% similarity, over the last 15 amino acids. Additionally, the proportion of out-of-frame proteins captured by such a chimeric binding domain would be expected to be as great, or greater, than the number of in frame proteins retained, which was not the case (see above).

The soybean LEA protein was, apparently, more discriminating than its Arabidopsis homologue, retrieving clones encoding only 4 different proteins from SL1 and clones encoding 4 in-frame, but different, proteins from SL2. The number of clones retrieved for in-frame proteins for the Arabidopsis LEA protein for SL1 and 2 were 10 and 9, respectively (Figure 3a). In all eight pans, multiple independent clones encoding portions of the same protein were recovered for 3/10, 2/4, 1/9, and 1/4 LEA protein interactors (Figure 3b–e). Pans conducted with SL1 retained phage with clones encoding portions of the same protein at both 25 °C and 41 °C for both LEA proteins (Figure 3b,c). Additionally, the two different LEA proteins enriched for phage with clones encoding portions of the same protein (AT1G15280) in four different pans, twice at 25 °C and twice at 41 °C (Figure 3b–d). The two LEA proteins also recovered clones encoding portions of protein AT1G30610 on four separate occasions, all from SL1 in pans at both temperatures (Figure 3b–c).

**Figure 4:**
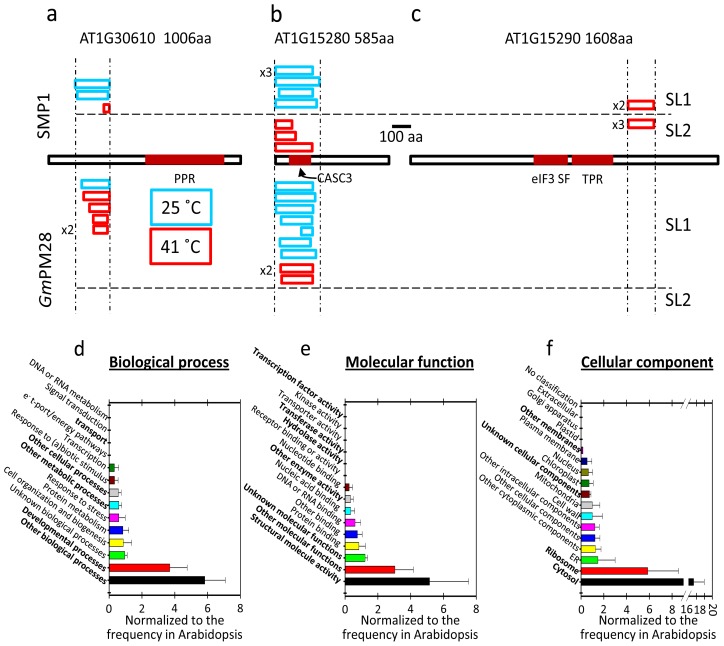
The three proteins recovered as independent clones among libraries, temperatures, and/or baits. (**a**) AT1G30610; (**b**) AT1G15280 and; (**c**) AT1G15290 are drawn to scale. Above these depictions, the size and location of the region of the protein encoded by each clone recovered by SMP1 is displayed. Below the depiction, clones recovered by *Gm*PM28 are displayed. Clones from SL1 are above the horizontal dashed lines, SL2 are below these lines. Scale bar is 100 aa. The color of the margin surrounding each clone was selected based on the temperature at which the clone(s) was/were isolated. GO (gene ontology) terms of the potential LEA protein interactors were categorized for biological processes (**d**); molecular function (**e**); and cellular component (**f**). The estimated frequencies of representation, relative to the whole Arabidopsis proteome, were boot-strapped to calculate deviations that were significantly greater or lesser than expected (bold text in each category).

The recovery of clones encoding identical proteins by both LEA proteins also provides insight into the specificity of interaction of these two LEA proteins. Instead of indiscriminate “spacer” or “shield” molecules, these two LEA proteins appear to have enhanced affinity for specific target proteins, despite the cross-species nature of the biopanning for the *Gm*PM28 (Figure 3a–e). Moreover, the portions of these proteins (AT1G15280 and AT1G30610) encoded by the different clones recovered by both SMP1 and *Gm*PM28 were similar (Figure 4a,b), while the region of AT1G15290 recovered by SMP1 from both SLs was also the same (Figure 4c). Because the libraries were constructed using random primers, this is unlikely to be due to any inherent bias in the libraries [28]. This is emphasized by the fact that the clones recovered for AT1G15280 were toward the extreme amino terminus, those for AT1G30610 were toward the amino 1/3 of the protein while those for AT1G15290 were centered toward the carboxy 1/3 of the protein (Figure 4a–c). The location of the conserved domain of each protein is colored dark red (AT1G30610: Pentatricopeptide repeat, PPR; AT1G15280: Cancer susceptibility candidate3, CASC3/Barentsz/eukaryotic translation initiation factor 4AIII binding protein, eIF4AIII; AT1G15290: eukaryotic translation initiation factor3 super family, eIF3 SF/Tetratricopeptide repeat, TPR), but there was no correlation between the region of the protein recovered in the pans and the identifiable domain(s) occurring in each protein (Figure 4a–c). However, there was a tendency for the regions recovered to be the most, or among the most, hydrophilic of the LEA protein interactor (Hopp/Woods analyses at ProtScale, [40], data not shown).

It is somewhat mystifying to recover clones encoding the same protein at both the moderate and higher temperature. It is possible that covalent attachment to the microtiter plate well is conducive to the LEA proteins adopting their protective shape, regardless of temperature, or that the presence of specific protein targets invokes an induced change in the conformation of these LEA proteins to better accommodate their protein target.

### 2.6. Proteins Resident in the Cytoplasm and Associated with the Translational Machinery Are Over-Represented among the Retrieved Targets

A gene ontology (GO) categorization of protein hits, relative to biological function, revealed that proteins involved in “developmental” and “other” processes (Figure 4d) were over represented. The most prominent molecular function listed was “structural”, as well as proteins belonging to “other” and “unknown functions” (Figure 4e). The cellular component to which the hits mostly belonged was the “cytosol”, particularly associated with the “ribosome” followed by “unknown” and “unknown membrane” components (Figure 4f). The cytosolic component to which many of the hits belong is encouraging considering that the SMP1 and *Gm*PM28 proteins are predicted to be without a transit peptide. Predictions of the subcellular compartment to which the two LEA proteins belong includes the cytoplasm (TAIR) and for SMP1, the Arabidopsis subcellular database (SUBA) predictions include the cytosol, nucleus, and mitochondrion [41].

The recovery of two proteins listed as EMB, “embryo-defective” (*i.e*., proteins essential for either gametogenesis, embryogenesis, or both) implies that these two LEA proteins have, as particular interactors, proteins whose activity is vital to cellular homeostasis (Table 1). The retention of phage displaying protions of a cyclophilin (petidyl-prolyl cis-trans isomerase; AT3G63400) suggests that protective proteins used to repair the stored proteome upon imbibition may be particular targets of these LEA proteins. Association with a LEA protein may allow the cyclophilin to remain inactive but functional in the dehydrated seed awaiting rehydration to commence the repair of proteins during the germination period that had suffered isomerization during quiescence [28]. It is reasonable to expect that such a protein would be a valuable asset the function of which would be worthy of protecting through interaction with LEA proteins during quiescence.

Both LEA proteins recovered proteins characterized as components of the ribosome. The proteins of the translational machinery have previously been hypothesized to be an Achilles heel of longevity in the mature dehydrated seed and may be preferential targets for repair following imbibtion [28]. This report further emphasizes that proteins of the translational machinery are afforded protection in the dehydrated seed through an association with, in this case, LEA proteins. The unifying concept suggested by these results is that components of the vital translational machinery are protected by at least some LEA proteins during the sojourn in the dehydrated state and are subsequently targets for repair after imbibition. The coordination of these protective mechanisms acting during (LEA proteins) and after (PIMT) the dehydrated period increases the opportunity for proteins associated with translation to remain functional, or regain function, upon imbibition.

An additional surmise is that because SMP1 and its soybean homologue *Gm*PM28 were both capable of retaining some of the same proteins, trans-species screens for LEA protein targets could be fruitful in the identification of heat-susceptible proteins in crop species using libraries of proteins from a model plant. However, based on results obtained with *Gm*PM28 with SL2 (no hits in common with either SL1 screened with *Gm*PM28, or with SMP1 for either library) results from such cross-species screens must be interpreted with caution.

The phage display using the SMP1 protein as bait was conducted to identify proteins that may require SMP1 to withstand 41 °C in order to invoke secondary seed dormancy, an adaptive trait that is compromised in the *smp1* mutant seeds of Col and Ler [28]. The protein recovered most frequently in the phage display (AT1G15280) is one about which little is known other than it undergoes phosphorylation [42]. The pentatricopeptide repeat containing protein (AT1G30610) is a lethal mutation in the homozygous state leading to defects in the suspensor [43,44]. Finally, the only report for protein AT1G15290 is that the transcription of the gene encoding it is altered during Cabbage Leaf Curly Virus infection [45]. With the exception of AT1G30610 (lethal in the homozygous state), the two LEA protein interactors AT1G15280 and AT1G15290 provide targets for reverse genetic analysis for their influence on secondary seed dormancy in Arabidopsis.

## 3. Experimental Section

### 3.1. Cloning SMP1 and *Gm*PM28

Amplification of the *SEED MATURATION PROTEIN1* (*SMP1*; intronless) and *Glycine max PM28* (Gm*PM28*) coding regions used *Arabidopsis thaliana* genomic DNA and soybean (*Glycine max*) reverse transcribed, mature dry seed, total RNA, respectively, as template. Amplicons were obtained using the PCR with Easy-A (Agilent Technologies, Inc., Santa Clara, CA, USA) DNA polymerase and gene specific primers (International DNA Technologies, Coralville, IA, USA) with non-homologous NdeI/SalI (*SMP1*) (5′-*CATATG*GCGAAGAACAAGGACGAC-3′; 5′-TT*GTC GAC*GCGGGGACTATCGTCTGTACG-3′) and NdeI/XhoI (Gm*PM28*) (5′-A*CATATG*GCGAAGAGC AAGGAAGAC-3′; 5′-T*CTCGAG*TCCAGGAGCTCCAGTGGTG-3′) restriction endonuclease sites engineered into the forward or reverse primer, respectively. Amplicons were cloned into a TA cloning vector, sequenced in both directions using the T7 or T3 promoter primers (5′-AATTA ATACGACTCACTATAG-3′; 5′-ATTAACCCTCACTAAAG-3′) then removed using the appropriate restriction endonucleases, gel-purified, and subcloned into *Nde*I and *Xho*I sites in pre-cleaved pET23b.

### 3.2. Recombinant Protein Production and Purification

SMP1 and *Gm*PM28 were produced from their respective pET23b plasmids in BL21(DE3)RIL (Strategene) cells by inoculating 1 L LB (100 μg·mL^−1^ ampicillin, 34 μg·mL^−1^ chloramphenicol) with 5 mL starter cultures. Un-induced cells were grown at 37 °C for circa 12 h before they were harvested. Cells were centrifuged and the spent media removed from the pellet which was then resuspended in 10 mL 10 mM Tris-HCl, pH 7.5. Lysozyme (1 mg) was added to the cells and, after 3 freeze-thaw cycles, the lysate was treated with 500 U Benzonase on ice. The lysate was centrifuged and the supernatant removed and introduced onto a nickel charged, pre-washed, Hi-Trap (Novagen) column. The column was attached to an FPLC (LKB Broma) washed extensively with 10 mM Tris-HCl buffer, pH 7.5 until the OD_280_ stabilized before a linear gradient of imidazole was introduced onto the column while 1 mL fractions were collected. Aliquots from these fractions were run using SDS-PAGE (15% total acrylamide) and fractions assessed for those containing the preponderance of the recombinant, hexahistidyl tagged proteins. These fractions were: (1) evaluated for protein purity and; (2) combined and then; (3) dialysed against 2 changes of 1000× volume of 10 mM Tris-HCl, pH 7.5. Protein concentration was ascertained colormetrically [46] (DC Protein Assay, Bio-Rad Laboratories, Hercules, CA, USA) and aliquots of the purified, dialysed protein were prepared and snap frozen in liquid nitrogen before being stored at −20 °C.

### 3.3. Characterization of SMP1 and *Gm*PM28 as Boiling-Stable Proteins

Aliquots of *E. coli* cells that had expressed SMP1 were heated at 60, 80, or 100 °C for 10 min before being centrifuged (30,000 × g, 20 min, 4 °C) and the supernatant recovered. The supernatant or unheated *E. coli* cells were frozen for 12 h at −20 °C. The next day the experiment was repeated and, following the introduction of SDS loading dye and boiling, the supernatants were loaded on 15% acrylamide gels and run. Additionally, upon purification over nickel columns, aliquots of the SMP1 and the *Gm*PM28 were boiled for 10 min, plunged on ice and centrifuged. The supernatant was removed, equal. volumes of 2× SDS-loading buffer added, the samples boiled 10 min and loaded on, and run through, 15% SDS-PAGE gels. Finally, 10 μL aliquots of nickel-affinity-purified *Gm*PM28 were held on ice or subjected to 60, 80, or 100 °C for 10 min before being centrifuged (30,000 × g, 20 min, 4 °C) and the supernatants recovered. The supernatants were run on 15% SDS-polyacrylamide gels.

### 3.4. Determination of Recombinant SMP1 and *Gm*PM28 Capacity to Bind to Microtiter Plate Wells

Dilution series of recombinant proteins, BSA, or buffer were introduced into the bottom of microtiter plate wells overnight at 4 °C in 10 mM Tris-HCl, pH 7.5 containing buffer or 10 mM sodium carbonate containing buffer at pH 9.6 or pH 10.6. The wells were washed, blocked, and monoclonal pentahistidine antibody (Qiagen Inc., Valencia, CA, USA) in 5% (w/v) blocking reagent (EMD Chemicals Inc., Gibbstown, NJ, USA) introduced into the wells. Following 2 h incubation, wells were washed extensively and secondary antibody (goat anti-mouse conjugated to alkaline phosphatase; Sigma-Aldrich Corp., St. Louis, MO, USA) in 5% (w/v) blocking reagent (EMD Chemicals Inc.) was added to each well for 1 h at 23 °C. The wells were washed and 100 μL of *para*-nitrophenylphosphate (Sigma-Aldrich Corp., St. Louis, MO, USA) was added to each well at 23 °C and, following 30 min color development, the reaction was stopped by the addition of 50 μL 3N NaOH and the wells were read at A_405_ using a Uniskan I ELISA plate reader (Labsystem and Flow Laboratories, Helsinki, Finland).

### 3.5. GLUCOSE 6 PHOSPHATE DEHYDROGENASE (G6PDH) Protection Assays

Recombinant LEA protein was tested for biological activity by co-incubation with the labile enzyme GLUCOSE 6 PHOSPHATE DEHYDROGENASE (G6PDH; EC 1.1.1.49). Additionally, equimolar concentrations of BSA, a protein known to protect G6PDH activity [39] or the commercial blocking reagent (EMD Chemicals Inc., Gibbstown, NJ, USA), used to coat the wells prior to panning, were tested for their capacities to protect G6PDH. Three units of G6PDH (144 pmol) or the same volume of water (neg. control) were added to 500 μL 10 mM Tris-HCl, pH 7.5 which did or did not contain (10 μg·mL^−1^) recombinant SMP1, *Gm*PM28, the molar equivalent to SMP1 of commercial BSA, or commercial blocking reagent (EMD Chemicals Inc.) (all 530 pmol, 3.6 molar excess of G6PDH). One aliquot was kept at room temperature for 1 h while a second was heated to 41 °C for 1 h. Additional controls constituted 1 h heated G6PDH to which recombinant LEA protein, BSA, or block (EMD Chemicals Inc.) was then added after heating which was either: (i) immediately analysed or; (ii) heated for an additional 1 h before being analysed. The recombinant LEA proteins, BSA, blocking reagent and water were all tested without G6PDH to verify that they were inactive in the G6PDH assay.

Following treatment, 10 μL (0.06 units) of G6PDH (or the water neg. control) was introduced into 90 μL which brought the 100 μL assay solution to a final concentration of reagents of: 50 mM glycylglycine; 2 mM d-glucose-6-phosphate; 0.67 mM β-nicotinamide adenine dinucleotide phosphate (NADP); and 10 mM MgCl_2_ at a pH of 7.4. The solution was pumped to mix and the cuvette placed in the spectrophotometer and a 6 min assay conducted at 25 °C at A_340_ [47]. The slopes of the increase in absorbance over a linear portion of the time course were calculated for each replication, the average slope was estimated and reported as a percentage of the G6PDH activity of the unheated enzyme. This was done for recombinant LEA proteins, BSA, water, and block (EMD Chemicals Inc.) in solution or when 10 μg·mL^−1^ of recombinant LEA proteins, BSA or block (EMD Chemicals Inc.) was placed in the bottom of microtiter plate wells overnight prior to washing, blocking with commercial blocking reagent (EMD Chemicals Inc.), and the introduction of G6PDH.

### 3.6. Biopanning

For each round of biopanning, two 96-well microtiter plates (clear, flat-bottom, standard tissue culture surface; Corning Incorporated, Corning, NY, USA) were marked with permanent ink, as either “41 °C” or “25 °C”. Both plates were washed extensively with water before 10 μg·mL^−1^ of: (a) recombinant SMP1; (b) recombinant *Gm*PM28 or; (c) purchased BSA (Sigma-Aldrich Corp. St. Louis, MO, USA), all in 100 μL of Tris, pH 7.5, were added to a well for each protein and plate. The dishes were covered with plastic wrap and left overnight at 4 °C. Unbound protein was removed by washing 10 times with 200 μL each time of TBS pH 7.5 before blocking with 200 μL 5% (w/v) blocking reagent (protein assay estimated 4 mg protein per well; EMD Chemicals Inc., Gibbstown, NJ, USA) in TBS for 1 h at room temperature, wrapped in plastic wrap. Excess block was removed by washing the wells 10 times with 200 μL each time of TBST before introducing the phage (100 μL) into each of the wells containing protein (3 per plate). One additional well in the “41 °C” plate was filled with TBST to be used to monitor the plate temperature at the commencement of washing. The plates were re-sealed with plastic and the “41 °C” plate put at 41 °C for 1 h. The “25 °C” plate was placed at 25 °C for 1 h also wrapped in plastic wrap. After one hour, the plate at 41 °C was rapidly removed from the oven, unwrapped and placed directly on the heating block (Pierce Reacti-Therm heating module, Thermo-Fisher, Rockford, IL, USA). Into the TBST containing-well was placed the sensor of a digital thermometer (Thermo sensor, Thermo-Fisher) to ensure that the block maintained the well contents at 41 °C. Wells of these plates were washed with ten 200 μL aliquots of the appropriate wash solution (*i.e*., either with pre-warmed 41 °C TTBS or with 25 °C TTBS) with a 1 min interval between the introduction of the wash and emptying it from the plate in each instance. After the tenth wash, 200 μL of BLT5403 cells were placed in the bottom of each well. The plates were wrapped in plastic wrap and put at 37 °C for 20 min to allow any SMP1-/*Gm*PM28-/BSA-retained phage to infect the bacteria.

At the end of 20 min, the plates were unwrapped and three 10 μL aliquots of bacteria from each well were taken, placed in 990 μL LB media with 100 μg·μL^−1^ ampicillin for titering. The remaining 170 μL of BLT5403 bacteria in each well was added to 50 mL BLT5403 cells at between 0.6 and 1.0 OD_600_ in 0.5 L Erlenmeyer flasks which were returned to 37 °C with agitation for phage amplification and lysate production. Upon lysis, the culture was brought to 0.5 M NaCl and centrifuged at 8000 × g for 10 min. The supernatant was transferred to clean, labeled tubes, a few drops of chloroform added, and the lysate stored at 4 °C until the next round of panning.

### 3.7. Titering

Serial dilutions of the cells from each replicate were made and 100 μL of these dilutions added to 250 μL of BLT5404 cells. To these cells was added 3 mL of top agarose which was then quickly spread over 100 μg·μL^−1^ ampicillin containing LB plates. Dishes were incubated at 23 °C overnight and plaques counted the following day. The plaque forming units (PFU)·mL^−1^ were calculated from the dilution and titer was ascertained.

### 3.8. Plaque Isolation, PCR and Sequencing

At each biopanning round, 18 individual plaque were selected using a Pasteur pipet from titering plates of sufficient dilution to produce well separated plaque. The agar/top agarose core containing the plaque was introduced to 100 μL Tris-HCl pH 8.5 and vortexed prior to an 90 μL aliquot being retrieved, heated at 65 °C for 10 min and 3 μL used in PCR reactions with T7 up and down primers (5′-GGAGCTGTCGTATTCCAGTC-3′; 5′-AACCCCTCAAGACCCGTTTA-3′). The 18 PCR reactions were run on 1% (w/v) agarose gels and visualized using ethidium bromide and transillumination. In the final biopanning round, those amplicons that were not from empty vectors were sequenced (The BigDye^®^ Terminator v3.1 Cycle Sequencing Kit, ABI Prism; using a T7 phage forward primer 5′-TGCTAACTTCCAAGCGGACC-3′ [48]). The sequences were examined for the linker arms, the orientation of the clone determined, and the clone used in WU-BLAST (TAIR, [49]) to determine: (1) whether it was in-frame and, if so; (2) whether it encoded part(all) of a coding sequence and; (3) the identity of the encoded protein.

### 3.9. Statistical Analysis

ELISA results (the average of 3 replicated wells for each of 4 protein dilutions), the percent activity of G6PDH retained in the presence of protein relative to blocking reagent (the average of two replications per protective protein, water or blocking solution for both the solution- and well-bound-assays), and the alteration in phage titer between either recombinant LEA protein and BSA (the average of three replications per well and protein for each biopan), were all subjected to Student *t* test at alpha = 0.05. When protection of G6PDH activity was anticipated based on prior work [39], a one-tailed *t*-Test was applied.

## 4. Conclusions

Phage display has proved to be a versatile technique when metabolic poisons [28], target protein alterations [28], *in vitro* protein bait-ligand associations, or supra-optimal temperatures, are used as part of the screen. The last attribute permitted the repeated recovery of clones encoding overlapping portions of the same protein targets from two different libraries by two LEA protein homologues. Instead of being indiscriminate “molecular shields”, some LEA proteins apparently have target molecules for which they have a particular affinity. This is, to our knowledge, the first time LEA proteins have been shown to bind preferentially to specific protein substrates.

The LEA proteins tested here associated frequently with proteins of the translational apparatus according to a gene ontology characterization. We have previously suggested that the proteins required for translation are particularly important to protect from permanent inactivation during seed quiescence [28] and LEA protein association may be one mechanism safeguarding this group from irrevocable damage during quiescence in the dehydrated state.

Lesions in the gene encoding SMP1 resulted in a loss of secondary dormancy, presumably due to a heat-susceptible, SMP1-target protein being without protection from denaturation [28]. There are at least three SMP1-target proteins repeatedly recovered in the biopans, which provide targets to screen for influences on the heat induction of secondary seed dormancy although one of these is an embryo lethal in the homozygous state.

## Supplemental Information



## Figures and Tables

**Table 1:** In-frame hits for SMP1 and *Gm*PM28 from SL1 and SL2 at 25 °C and 41 °C. Hits: the total number of sequences encountered encoding this protein for this library, whether it was identical or not. Independent clones: those encoding the same protein but from different pans of the same library or different lengths cDNA.

In-Frame Hits of SMP1 at 25 °C Using SL1 after 4 Rounds of Panning				

Hits	Locus Identifier	Protein Identity	Independent Clones	Subcellular Residence/Authority
6	AT1G15280	CASC3/Barentsz eIF4AIII binding	4	Predictors-SubLoc: nucleus; WoLFPSORT: nucleus
2	AT1G30610	EMB88, EMB2279 pentatricopeptide (PPR) repeat-containing protein	2	Predictors-iPSORT: plastid; MitoPred: mitochondrion; MultiLoc: plastid; PeroxP: peroxisome; Predotar: plastid; SubLoc: nucleus; TargetP: plastid; WoLFPSORT: nucleus
1	AT1G58210	EMB1674 | kinase interacting family protein	1	Predictors-iPSORT: mitochondrion; MitoPred: mitochondrion; MultiLoc: plastid; Predotar: mitochondrion; SubLoc: nucleus; TargetP: plastid; WoLFPSORT: nucleus
1	AT3G55170	Ribosomal L29 family protein	1	**SUBA MS/MS:** cytosol: 17934214; plasma membrane: 19334764 Predictors: LOCtree: plastid; MitoPred: mitochondrion; SubLoc: nucleus; WoLFPSORT: nucleus

**In-Frame Hits of** ***Gm*PM28 at 25 °C Using SL1 after 4 Rounds of Panning**				

**Hits**	**Locus Identifier**	**Protein Identity**	**Independent Clones**	**Subcellular Residence/Authority**

7	AT1G15280	CASC3/Barentsz eIF4AIII binding	7	Predictors-SubLoc: nucleus; WoLFPSORT: nucleus
1	AT3G63400	Cyclophilin-like peptidyl-prolyl *cis*-*trans* isomerasefamily protein	1	Predictors-SubLoc: nucleus
1	AT1G30610	EMB88, EMB2279 | pentatricopeptide (PPR) repeat-containing protein	1	Predictors-iPSORT: plastid; MitoPred: mitochondrion; MultiLoc: plastid; PeroxP: peroxisome; Predotar: plastid; SubLoc: nucleus; TargetP: plastid; WoLFPSORT: nucleus
1	AT2G02160	CCCH-type zinc finger family protein	1	Predictors-MitoPred: mitochondrion; SubLoc: nucleus; WoLFPSORT: nucleus

**In-Frame Hits of SMP1 at 41 °C Using SL1 after 4 Rounds of Panning**				

**Hits**	**Locus Identifier**	**Protein Identity**	**Independent Clones**	**Subcellular Residence/Authority**

1	AT1G30610	EMB88, EMB2279 pentatricopeptide (PPR) repeat-containing protein	1	Predictors-iPSORT: plastid; MitoPred: mitochondrion; MultiLoc: plastid; PeroxP: peroxisome; Predotar: plastid; SubLoc: nucleus; TargetP: plastid; WoLFPSORT: nucleus
3	AT2G36640	ATECP63, ECP63 embryonic cell protein 63	2	Predictors-LOCtree: cytosol SubLoc: nucleus WoLFPSORT: peroxisome
1	AT1G55310	SR33, SCL33, At-SCL33 | SC35-like splicing factor	1	Predictors-interchromatin granule, nuclear speck, nucleolus, plasma membrane/TAIR
1	AT5G46430	Ribosomal protein L32e	1	**SUBA MS/MS:** cytosol: 17934214; cytosol: 21166475; plasma membrane: 19334764; plasma membrane: 15574830; Predictors-iPSORT: mitochondrion; LOCtree: mitochondrion; MitoPred: mitochondrion; Mitoprot 2: mitochondrion; MultiLoc: plastid; Predotar: mitochondrion; SubLoc: mitochondrion; TargetP: mitochondrion; WoLFPSORT: cytosol
2	AT1G15290	Tetratricopeptide repeat (TPR)-like superfamily	1	**SUBA MS/MS:** cytosol: 21166475; Predictors-SubLoc: nucleus; WoLFPSORT: nucleus
1	AT2G31410	unknown protein	1	Predictors-MitoPred: mitochondrion; SubLoc: nucleus; WoLFPSORT: nucleus
1	AT1G16610	SR45, RNPS1 | arginine/serine-rich 45	1	Mitochondria, nucleus plastid/BAR Cell eFP Browser

**In-Frame Hits of** ***Gm*PM28 at 41 °C Using SL1 after 4 Rounds of Panning**				

**Hits**	**Locus Identifier**	**Protein Identity**	**Independent Clones**	**Subcellular Residence/Authority**

5	AT1G30610.1	EMB88, EMB2279 | pentatricopeptide (PPR)repeatcontaining protein	4	Predictors-iPSORT: plastid; MitoPred: mitochondrion; MultiLoc: plastid; PeroxP: peroxisome; Predotar: plastid; SubLoc: nucleus; TargetP: plastid; WoLFPSORT: nucleus
3	AT1G15280.1	CASC3/Barentsz eIF4AIII binding	2	Predictors-SubLoc: nucleus; WoLFPSORT: nucleus

**In-Frame Hits of SMP1 at 25 °C Using SL2 after 4 Rounds of Panning**				

**Hits**	**Locus Identifier**	**Protein Identity**	**Independent Clones**	**Subcellular Residence/Authority**

2	AT1G11630	Pentatricopeptide repeat-containing protein	2	**SUBA MS/MS:** mitochondrion: 14671022; Predictors-iPSORT: mitochondrion; LOCtree: mitochondrion; MitoPred: mitochondrion; MultiLoc: plastid; Predotar : mitochondrion; SubLoc : nucleus; TargetP : mitochondrion; WoLFPSORT : plastid
3	AT1G65090	unknown protein	3	cytosol, mitochondria/BAR Cell eFP Browser

**In-Frame Hits of** ***Gm*PM28 at 25 °C Using SL2 after 4 Rounds of Panning**				

**Hits**	**Locus Identifier**	**Protein Identity**	**Independent Clones**	**Subcellular Residence/Authority**

8	AT5G44120	CRA1, ATCRA1, CRU1 | RmlC-like cupins superfamily	1	**SUBA MS/MS**: plasma membrane; Predictors-Predotar: endoplasmic reticulum; SubLoc: nucleus; TargetP: extracellular; WoLFPSORT: vacuole

**In-Frame Hits of SMP1 at 41 °C Using SL2 after 4 Rounds of Panning**				

**Hits**	**Locus Identifier**	**Protein Identity**	**Independent Clones**	**Subcellular Residence/Authority**

3	AT1G15280.2	CASC3/Barentsz eIF4AIII binding	3	Predictors-SubLoc: nucleus; WoLFPSORT: nucleus
3	AT1G15290.1	Tetratricopeptide repeat (TPR)-like superfamily	1	**SUBA MS/MS:** cytosol: 21166475; Predictors-SubLoc: nucleus; WoLFPSORT: nucleus
1	AT4G12400.2	Hop3|stress-inducible protein, putative	1	No information
1	AT1G54870.1	NAD(P)-binding Rossmann-fold superfamily protein	1	Predictors-MultiLoc: mitochondrion; SubLoc: cytosol; WoLFPSORT: plastid
1	AT3G58680.1	MBF1B, ATMBF1B | multiprotein bridging factor 1B	1	**GFP:** cytosol: 15610358; nucleus: 15610358; Annotators-AmiGO: nucleus; Predictors-LOCtree: cytosol; MitoPred: mitochondrion; Predotar: plastid; SubLoc: nucleus; WoLFPSORT: nucleus
1	AT3G01690.1	alpha/beta-Hydrolases superfamily protein	1	Predictors-iPSORT: mitochondrion; SubLoc: nucleus; TargetP: plastid; WoLFPSORT: cytosol
1	AT5G52240.1	MSBP1, ATMP1, AtMAPR5 membrane steroid binding protein1	1	**SUBA MS/MS:** endoplasmic reticulum: 16618929; plasma membrane: 17317660; plasma membrane: 19334764; plasma membrane: 17644812; Annotators-AmiGO: plasma membrane; Predictors-SubLoc: cytosol; TargetP: extracellular; WoLFPSORT: endoplasmic reticulum

**In-Frame Hits of** ***Gm*PM28 at 41 °C Using SL2 after 4 Rounds of Panning**				

**Hits**	**Locus Identifier**	**Protein Identity**	**Independent Clones**	**Subcellular Residence/Authority**

4	AT5G18380	Ribosomal protein S5 domain 2-like superfamily	3	Annotators-UniProt : cytosol Predictors-iPSORT: plastid; LOCtree: cytosol; Mitoprot 2: mitochondrion; SubLoc: mitochondrion; WoLFPSORT: cytosol
2	AT4G04470	PMP22| Peroxisomal membrane 22 kDa (Mpv17/PMP22) family protein	1	**SUBA MS/MS:** peroxisome: 18931141; plasma membrane: 19334764; Annotators-TAIR : peroxisome; AmiGO: peroxisome Predictors-iPSORT: mitochondrion; LOCtree: mitochondrion; MitoPred: mitochondrion; Mitoprot 2: mitochondrion; MultiLoc: mitochondrion; PeroxP: no data; Predotar: no data; SubLoc: mitochondrion; TargetP: mitochondrion; WoLFPSORT: plastid
1	AT2G19540	Transducin family protein/WD-40 repeat family protein	1	Predictors-SubLoc: cytosol; WoLFPSORT: mitochondrion
