# Supplementary material for: Identification of Late Embryogenesis Abundant (LEA) Protein Putative Interactors Using Phage Display
Source: Int J Mol Sci. 2012 May 29;13(6):6582–603. doi: 10.3390/ijms13066582 (PMC3397483; doi:10.3390/ijms13066582)

Identification of Late Embryogenesis Abundant (LEA) Protein Putative Interactors Using Phage Display

Supplemental Information

Figure S.1. The percentage (%) of randomly chosen plaque that contained insert greater than 300 bp from seed library 1 (SL1) for SMP1- (**a** and **d**), *Gm*PM28- (**b** and **e**), and BSA-biopans (**c** and **f**) for each round of biopanning at either 25 or 41 ℃.

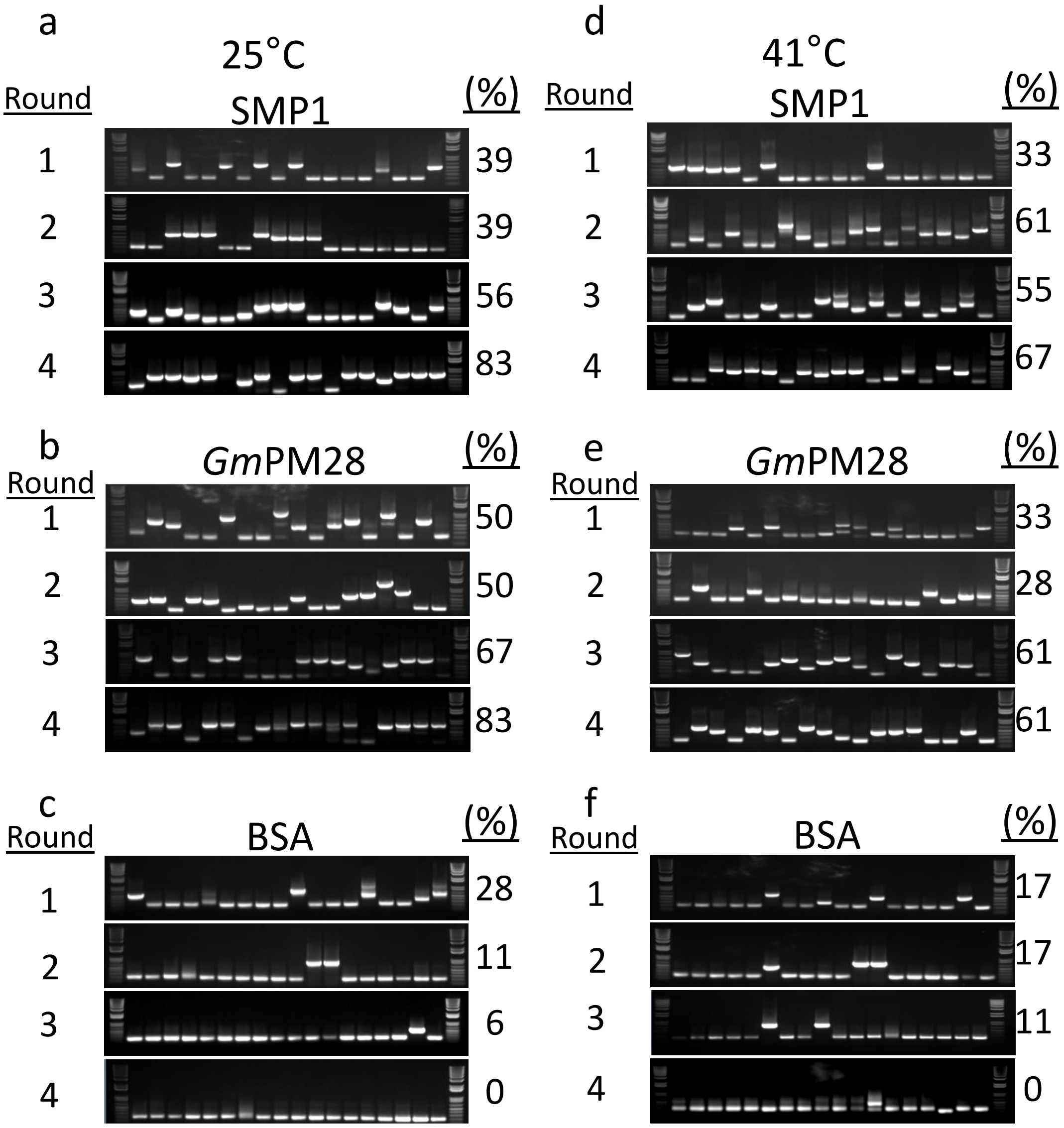

Figure S.2. The percentage (%) of randomly chosen plaque that contained insert greater than 300 bp from seed library 2 (SL2) for SMP1- (**a** and **d**), *Gm*PM28- (**b** and **e**), and BSA-biopans (**c** and **f**) for each round of biopanning at either 25 or 41 ℃.

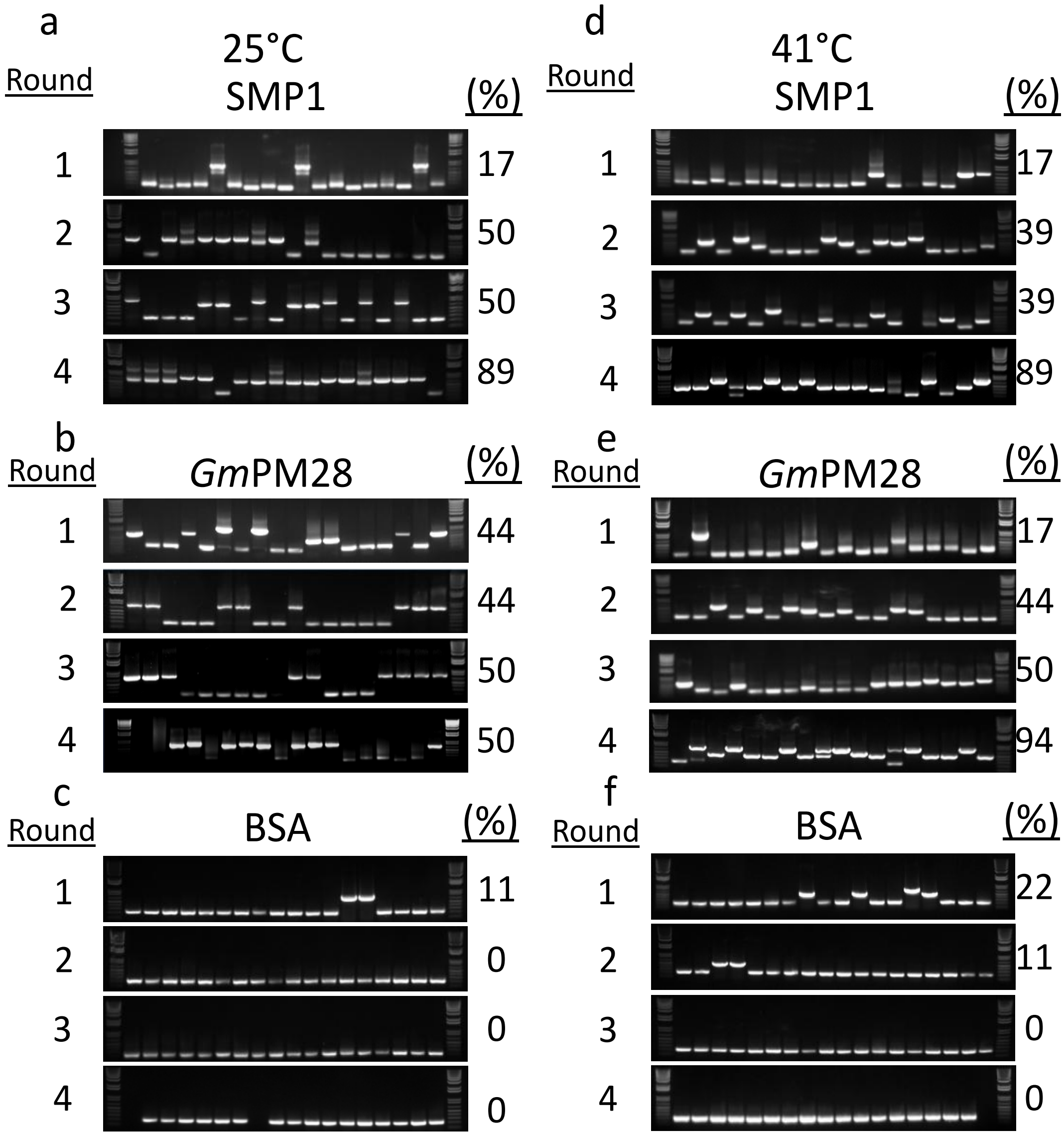

Supplement: Supplementary file 1 [file ijms-13-06582-s001.pdf]
